# Learning of Precise Spike Times with Homeostatic Membrane Potential Dependent Synaptic Plasticity

**DOI:** 10.1371/journal.pone.0148948

**Published:** 2016-02-22

**Authors:** Christian Albers, Maren Westkott, Klaus Pawelzik

**Affiliations:** Institute for Theoretical Physics, University of Bremen, Bremen, Germany; McGill University, CANADA

## Abstract

Precise spatio-temporal patterns of neuronal action potentials underly e.g. sensory representations and control of muscle activities. However, it is not known how the synaptic efficacies in the neuronal networks of the brain adapt such that they can reliably generate spikes at specific points in time. Existing activity-dependent plasticity rules like Spike-Timing-Dependent Plasticity are agnostic to the goal of learning spike times. On the other hand, the existing formal and supervised learning algorithms perform a temporally precise comparison of projected activity with the target, but there is no known biologically plausible implementation of this comparison. Here, we propose a simple and local unsupervised synaptic plasticity mechanism that is derived from the requirement of a balanced membrane potential. Since the relevant signal for synaptic change is the postsynaptic voltage rather than spike times, we call the plasticity rule Membrane Potential Dependent Plasticity (MPDP). Combining our plasticity mechanism with spike after-hyperpolarization causes a sensitivity of synaptic change to pre- and postsynaptic spike times which can reproduce Hebbian spike timing dependent plasticity for inhibitory synapses as was found in experiments. In addition, the sensitivity of MPDP to the time course of the voltage when generating a spike allows MPDP to distinguish between weak (spurious) and strong (teacher) spikes, which therefore provides a neuronal basis for the comparison of actual and target activity. For spatio-temporal input spike patterns our conceptually simple plasticity rule achieves a surprisingly high storage capacity for spike associations. The sensitivity of the MPDP to the subthreshold membrane potential during training allows robust memory retrieval after learning even in the presence of activity corrupted by noise. We propose that MPDP represents a biophysically plausible mechanism to learn temporal target activity patterns.

## Introduction

Precise and recurring spatio-temporal patterns of action potentials are observed in various biological neuronal networks. In zebra finches, precise sequences of activations in region HVC are found during singing and listening to the own song [1]. Also, when spike times of sensory neurons are measured, the variability of latencies relative to the onset of a externally induced stimulus is often higher than if the latencies are measured relative to other sensory neurons [2, 3]; spike times covary. Therefore, information about the stimulus is coded in spatio-temporal spike patterns. Theoretical considerations show that in some situations spike-time coding is superior to rate coding [4]. Xu and colleagues demonstrated that through associative training it is possible to imprint new sequences of activations in visual cortex [5], which shows that there are plasticity mechanisms which are used to learn precise sequences.

These observations suggest that spatio-temporal patterns of spike activities underlie coding and processing of information in many networks of the brain. However, it is not known which synaptic plasticity mechanisms enable neuronal networks to learn, generate, and read out precise action potential patterns. A theoretical framework to investigate this question is the chronotron, where the postsynaptic neuron is trained to fire a spike at predefined times relative to the onset of a fixed input pattern [6]. A natural candidate plasticity rule for chronotron training is Spike-Timing Dependent Plasticity (STDP) [7] in combination with a supervisor who enforces spikes at the desired times. Legenstein and colleagues [8] investigated the capabilities of supervised STDP in the chronotron task and identified a key problem: STDP has no means to distinguish between desired spikes caused by the supervisor and spurious spikes resulting from the neuronal dynamics. As a result every spike gets reinforced, and plasticity does not terminate when the correct output is achieved, which eventually unlearns the desired synaptic state. The failings of STDP hint at the requirements of a working learning algorithm. Information about the type of a spike (desired or spurious) has to be available to each synapse, where it modulates spike time based synaptic plasticity. Synapses evoking undesired spikes should be weakened, synapses that contribute to desired spikes should be strengthened, but only until the self-generated output activity matches the desired one. Plasticity should cease if the output neurons generate the desired spikes without supervisor intervention. In other words, at the core of a learning algorithm has to be a comparison of actual and target activity, and synaptic changes have to be computed based on the difference between the two.

In recent years, a number of supervised learning rules have been proposed to train to fire temporally precise output spikes in response to recurring spatio-temporal input patterns [6, 9–11]. They compare the target spike train to the self-generated (actual) output and devise synaptic changes to transform the latter into the former. Another group of algorithms performs a comparison of actual and target firing rate instead of spike times [12–15]. Because they work with the instaneous firing rate, they do not rely on sampling of discrete spikes and therefore the comparison is local in time. It is interesting to note that these learning algorithms are implicitely sensitive to the current membrane potential, of which the firing rate is a monotonous function. These algorithms are useful to probe the capabilities of neuronal networks, but when it comes to biological implementation, there are several open questions. Some of the algorithms (e.g. [6, 11]) rely on explicit feedback about the error on the output and precise instructions on how to change input weights from a supervisor of undefined nature. It is not clear how such a supervisor could be realized, nor how it computes the error and instructions and relays them to the neuron. Other algorithms (e.g. [10, 12, 14]) use a more implicit supervisor or teacher who induces spike-like signals for associative learning, while trying to unlearn self-generated activity. Similarly, the nature of these signals is not clear. They can not be spikes, since those are stereotypical signals: Spikes have no color. From these observations we can deduce the requirements for a biologically plausible learning rule: It should be local in the sense that it does not need external feedback about the error on the output. Also, ideally the synapse is only sensitive to signals that are visible in the neuronal dynamics.

In this study, we investigate the learning capabilities of a plasticity rule which relies only on postsynaptic membrane potential and presynaptic spikes as signals. To distinguish it from spike times based rules, we call it Membrane Potential Dependent Plasticity (MPDP). We derive MPDP from a homeostatic requirement on the voltage and show that in combination with spike after-hyperpolarisation (SAHP) it is compatible with experimentally observed STDP of inhibitory synapses [16]. Despite its Anti-Hebbian nature, MPDP combined with SAHP can be used to train a neuron to generate desired temporally structured spiking output in an associative manner. During learning, the supervisor or teacher induces spikes at the desired times by a strong input. Because of the differences in the time course of the voltage, a synapse can sense the difference between spurious spikes caused by weak inputs and teacher spikes caused by strong inputs. As a consequence, weight changes are matched to the respective spike type. Therefore, our learning algorithm provides a biologically plausible answer for the open question presented above. Additionally, the sensitivity of MPDP to subthreshold voltage leads to a noise-tolerant network after training with noise free examples. For a quantitative analysis, we simplify the neuron model and apply our learning mechanism to train a Chronotron [6]. We find that the attainable memory capacity is comparable to that of a range of existing learning rules [6, 10, 11], however the noise tolerance after training is superior in networks trained with MPDP in comparison to those trained with the other learning algorithms.

## Materials and Methods

In this section, we present the models used. We start with the simpler leaky integrate-and-fire neuron model (LIF neuron) and use it to derive the MPDP rule. We then show how MPDP can be applied to a more realistic conductance based integrate-and-fire neuron. Last, we describe the Chronotron setup we use for quantitatively assessing the memory capacity of MPDP.

### The LIF neuron and derivation of MPDP

**Fig 1:**
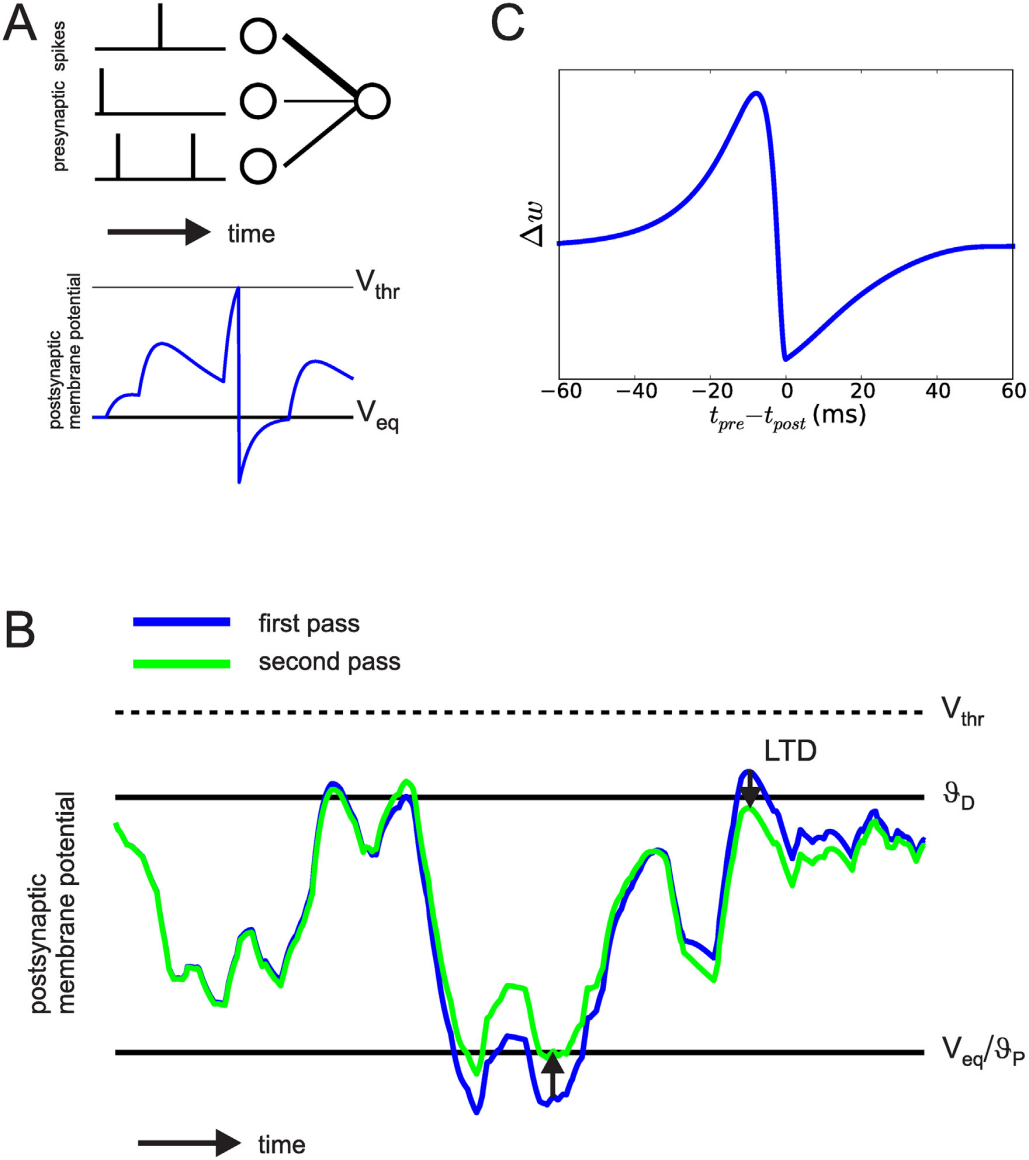
**A:** The model network has a simple feed-forward structure. The top picture shows three pre- and one postsynaptic neurons, connected by synapses. Line width in this example corresponds to synaptic strength. Bottom picture shows the postsynaptic membrane potential in response to the input. **B**: Illustration of Anti-Hebbian Membrane Potential Dependent Plasticity (MPDP). A LIF neuron is presented twice with the same presynaptic input pattern. Excitation never exceeds *V*_*thr*_. MPDP changes synapses to counteract hyperpolarization and depolarization occuring in the first presentation (blue trace), reducing (arrows) them on the second presentation (green trace). **C**: Homeostatic MPDP on inhibitory synapses is compatible with STDP as found in experiments. Weight change is tested for different temporal distances between pre- and postsynaptic spiking, with the presynaptic neuron being an inhibitory neuron. Δ*w* here denotes the change of the increase in conductance in an inhibitory synapse upon a presynaptic spike. The resulting spike timing characteristic is in agreement with experimental data on STDP of inhibitory synapses [16]. Note that an increase of the weight leads to a suppressive effect on the membrane potential.

We investigated plasticity processes in a simple single-layered feed-forward network with *N* (presynaptic) input neurons and one (postsynaptic) output neuron (see Fig 1A). For the input population we stochastically generate spatio-temporal spike patterns which are kept fixed throughout training (frozen noise). We denote the time of the *k*-th spike of presynaptic neuron with index *i* as $t_{i}^{k}$.

The postsynaptic neuron is modeled as a LIF neuron. The evolution of the voltage *V*(*t*) over time is given by

$$\begin{array}{c} \tau_{m}\dot{V}=-V+I_{syn}+I_{ext}\;. \end{array}$$ (1)

*I*_*syn*_ and *I*_*ext*_ are synaptic and external currents, respectively, and *τ*_*m*_ is the membrane time constant of the neuron. If the voltage reaches the firing threshold *V*_*thr*_ at time *t*_*post*_, the neuron generates a spike and undergoes immediate reset to the reset potential *V*_*reset*_ < 0. In the absence of any input currents, the neuron relaxes to an equilibrium potential of *V*_*eq*_ = 0. Synaptic currents are given by

$$\begin{array}{c} \tau_{s}{\dot{I}}_{syn}=-I_{syn}+\sum_{i}w_{i}\sum_{k}\delta \left(t-t_{i}^{k}\right)\;. \end{array}$$ (2)

*τ*_*s*_ is the decay time constant of synaptic currents and *w*_*i*_ is the synaptic weight of presynaptic neuron *i*. For ease of derivation of MPDP, we reformulated the LIF model. Because of the linearity of Eq 1, we can write the voltage as the sum of kernels for postsynaptic potentials (PSPs) *ε*(*s*) and resets *R*(*s*):

$$\begin{array}{c} V\left(t\right)=\sum_{i}w_{i}\sum_{k}\varepsilon \left(t-t_{k}^{i}\right)+\sum_{t_{post}}R\left(t-t_{post}\right)+\int_{0}^{\infty }\kappa \left(t-s\right)I_{ext}\left(s\right)ds\;. \end{array}$$ (3)

*κ* = exp(−(*t* − *s*)/*τ*_*m*_) is the passive response kernel by which external currents are filtered. The other kernels are

$$\begin{array}{l} \varepsilon \left(s\right)\;=\;\Theta \left(s\right)\frac{1}{\tau_{m}-\tau_{s}}\left(\text{exp}\left(-s/\tau_{m}\right)-\text{exp}\left(-s/\tau_{s}\right)\right)\\ R\left(s\right)\;=\;\Theta \left(s\right)\left(V_{reset}-V_{thr}\right)\text{exp}\left(-s/\tau_{m}\right). \end{array}$$ (4)

Θ(*s*) is the Heaviside step function. This formulation is also known as the simple Spike Response Model (SRM_0_, [17]).

We next derive the plasticity rule from the naive demand of a balanced membrane potential: The neuron should not be hyperpolarized nor too strongly depolarized. This is a sensible demand for the dynamics of a neuronal network, because it holds the neurons at sensitive working points and keeps metabolic costs down. For the formalization of the objective, we introduce an error function which assigns a value to the current voltage:

$$\begin{array}{c} 2E\left(V\left(t\right)\right)=\gamma {\left({\left[V\left(t\right)-\vartheta_{D}\right]}_{+}\right)}^{2}+{\left({\left[\vartheta_{P}-V\left(t\right)\right]}_{+}\right)}^{2}\;, \end{array}$$ (5)

where *ϑ*_*D*, *P*_ are thresholds for plasticity, and *γ* is a factor that scales synaptic long-term depression (LTD) and long-term potentiation (LTP) relative to each other. [.]_+_ denotes the rectifying bracket, i.e. [*x*]_+_ = *x* if *x* > 0 and zero else. Whenever *V*(*t*) > *ϑ*_*D*_ or *V*(*t*) < *ϑ*_*P*_, the error function is greater than zero. Therefore, to minimize the error, the voltage must stay between both thresholds. In this study, we choose *ϑ*_*P*_ = *V*_*eq*_. *ϑ*_*D*_ is set between the firing threshold and *V*_*eq*_. From the error function, a weight change rule can be obtained by computing the partial derivative of *E*(*t*) with respect to weight *w*_*i*_:

$$\begin{array}{c} \begin{array}{cc} \frac{\partial E(V(t))}{\partial w_{i}} & =\gamma {\left[V\left(t\right)-\vartheta_{D}\right]}_{+}\frac{\partial V(t)}{\partial w_{i}}-{\left[\vartheta_{P}-V\left(t\right)\right]}_{+}\frac{\partial V(t)}{\partial w_{i}}\\ & =\left(\gamma {\left[V\left(t\right)-\vartheta_{D}\right]}_{+}-{\left[\vartheta_{P}-V\left(t\right)\right]}_{+}\right)\sum_{k}\varepsilon \left(t-t_{i}^{k}\right)\;. \end{array} \end{array}$$ (6)

The MPDP rule then reads

$$\begin{array}{c} {\dot{w}}_{i}=-\eta \frac{\partial E(V(t))}{\partial w_{i}}=\eta \left(-\gamma {\left[V\left(t\right)-\vartheta_{D}\right]}_{+}+{\left[\vartheta_{P}-V\left(t\right)\right]}_{+}\right)\sum_{k}\varepsilon \left(t-t_{i}^{k}\right)\;. \end{array}$$ (7)

*η* is the learning rate. The weights change along the gradient of the error function, i.e. MPDP is a gradient descent rule that minimizes the error resulting from a given input pattern.

### The conductance based LIF neuron

The simple model above suffers from the fact MPDP is agnostic to the type of synapse. In principle, MPDP can turn excitatory synapses into inhibitory ones by changing the sign of any weight *w*_*i*_. Since this is a violation of Dale’s law, we present a more biologically realistic scenario involving MPDP. We split the presynaptic population into *N*_*e*_ excitatory and *N*_*i*_ inhibitory neurons. The postsynaptic neuron is modeled as a conductance based LIF neuron governed by

$$\begin{array}{c} C_{m}\frac{\text{d}V}{\text{d}t}=-g_{L}\left(V-V_{L}\right)-\left(g_{s}+g_{f}\right)\left(V-V_{h}\right)-g_{ex}\left(V-V_{ex}\right)-g_{in}\left(V-Vin\right)\;, \end{array}$$ (8)

where *V* denotes the membrane potential, *C*_*m*_ the membrane capacitance, *V*_*L*_ the resting potential, *g*_*L*_ the leak conductance, *V*_*i*_ and *V*_*ex*_ the reversal potential of inhibition and excitation, respectively and *g*_*in*_ and *g*_*ex*_ their respective conductances. The spike after-hyperpolarisation is modeled to be biphasic consisting of a fast and a slow part, described by conductances *g*_*f*_ and *g*_*s*_ that keep the membrane potential close to the hyperpolarisation potential *V*_*h*_. When the membrane potential surpasses the spiking threshhold *V*_*thr*_ at time *t*_*post*_, a spike is registered and the membrane potential is reset to *V*_*reset*_ = *V*_*h*_. All conductances are modeled as step and decay functions. The reset conductances are given by

$$\begin{array}{c} \tau_{f,s}{\dot{g}}_{f,s}=-g_{f,s}+\Delta g_{f,s}\sum_{t_{post}}\delta \left(t-t_{post}\right)\;, \end{array}$$ (9)

where Δ*g*_*f*, *s*_ is the increase of the fast and slow conductance at the time of each postsynaptic spike, respectively. They decay back with time constants *τ*_*f*_ < *τ*_*s*_. The input conductances *g*_*ex*_ and *g*_*in*_ are step and decay functions as well, that are increased by *w*_*i*_ when presynaptic neuron *i* spikes and decay with time constant *τ*_*s*_,

$$\begin{array}{c} \tau_{s}\frac{dg_{ex}}{dt}=-g_{ex}+\sum_{i\in exc}w_{i}\sum_{k}\delta \left(t-t_{i}^{k}\right)\;, \end{array}$$ (10)

for excitatory synapses and

$$\begin{array}{c} \tau_{s}\frac{dg_{in}}{dt}=-g_{in}+\sum_{j\in inh}w_{j}\sum_{k}\delta \left(t-t_{j}^{k}\right)\;f \end{array}$$ (11)

for inhibitory synapses. *w*_*i*, *j*_ denotes the strength of synapse *i*, *j*, which can be either in the set of excitatory synapses *i* ∈ *exc* or inhibitory synapses *j* ∈ *inh*.

In this model, we employ MPDP as defined by Eq 7 with the following restrictions:

- Technically, there is no fixed PSP kernel for the conductance based model, since the amplitude of a single PSP depends on the current voltage. Still, we use the same rule by keeping track of “virtual PSPs” given by the kernel *ε*(*s*), Eq 4 for each synapse that do not affect the neuronal dynamics.
- MPDP affects only inhibitory synapses. Excitatory ones are kept fixed.
- Because inhibitory synapses have negative impact on the neuron, we exchange LTP and LTD in the MPDP rule to account for that. Formally, we introduce thresholds $\vartheta_{D}^{I}$ and $\vartheta_{P}^{I}$.

As in the linear LIF neuron model, $\vartheta_{D}^{I}$ lies at the equilibrium potential *V*_*L*_, and an inhibitory synapse depresses whenever it is active and $V\left(t\right)<\vartheta_{D}^{I}$. Similarly, when $V\left(t\right)>\vartheta_{P}^{I}$, any active inhibitory synapse gets potentiated. Note that the qualitative effect on the membrane potential remains unchanged to the example in Fig 1B.

### Evaluation of memory capacity

The memory capacity of a typical neuronal network in a given task crucially depends on the learning rules applied (for an example in spiking networks see [6]). Recently, it was shown that the maximal number of spiking input-output associations learnable by a postsynaptic neuron lies in the range of 0.1 to 0.3 per presynaptic input neuron [11]. The exact number depends on a parameter $\tau =\sqrt{\tau_{m}\cdot \tau_{s}}$ of the neuron and the average postsynaptic firing rate. It was shown that the HTP algorithm has optimal capacity, and for small window of tolerance FP-Learning achieves similar capacity as the HTP method [11]. Here, we evaluate the memory capacity attainable with MPDP and compare it with ReSuMe [10], E-Learning [6] and FP-Learning [11]. We use the latter rule as a reference for maximal capacity, although our chosen window of tolerance is not small. For ease of comparison, we adapt the Chronotron setting introduced by Florian [6], use the simple neuron model of the LIF neuron and let synapses change their sign. The definitions of input-output associations between spatio-temporal spiking patterns and consequently the memory capacity is similar to the ones used in Tempotron and Perceptron training [18, 19]. We provide a short description of ReSuMe, E-Learning and FP-Learning below.

#### Chronotron setting

The goal of the Chronotron is to imprint input-output associations into the weights. One input pattern consists of spatio-temporal spiking activity of the *N* input neurons with duration *T* = 200*ms*. In each pattern, each input neuron spikes exactly once, with spike times $t_{i}^{\mu }$ drawn i.i.d. from the interval [0, *T*]. *μ* ∈ 1, …, *P* indexes the patterns. For each input pattern we draw one desired output spike time $t_{d}^{\mu }$ i.i.d. from the interval [Δ_*edge*_, *T* − Δ_*edge*_], with Δ_*edge*_ = 20*ms*. We reduce the length of the output interval to ensure that each output spike in principle can be generated by the input. If the desired output spike time is too early there might be no input spikes earlier than *t*_*d*_, which makes it impossible for the postsynaptic neuron to generate the desired output. After all *P* patterns have been generated, we keep them fixed for the duration of network training and recall testing. Training is organized in learning trials and learning blocks. A learning trial in MPDP consists of the presentation of one of the input patterns and concurrent induction of a teacher spike at time $t_{d}^{\mu }$ by injection of a supratheshold delta-pulse current into the postsynaptic neuron by the supervisor. In all other learning rules, the supervisor passively observes the output activity and changes weights afterwards based on the actual output. A learning block consists of *P* learning trials, with each of the different input patterns presented exactly once in randomized order. After each learning trial, synaptic weights are updated. After each learning block, we present the input patterns again to test the recall quality. Supervisor intervention and synaptic plasticity are switched off for recall trials.

#### Computing the capacity

We test the capacity of each learning rule (MPDP, ReSuMe, E-Learning and FP-Learning) by training networks of different sizes, *N* ∈ {200, 500, 1000, 2000}. Because we assume that the number of patterns or input-output associations that can be learned scales with *N* [6, 11], we introduce the load parameter *α* with *P* = *αN*. We pick discrete *α* ∈ [0.01, 0.3]. For each combination of *α* and *N*, we generate 50 different realizations of *P* patterns and *N* initial weights, which are drawn from a gaussian distribution with mean and standard deviation *T*⋅30*mV*/*N*. For a non-spiking neuron (i.e. Eq 1 with *V*_*thr*_ ≫ 1) this would result in an average membrane potential of 30*mV* before learning. As a result initially the postsynaptic neuron fires several spurious spikes. This way we test the ability of a learning rule to extinguish them.

After each learning block, the recall is tested. Recall is counted as a success if the postsynaptic neuron fires exactly one output spike in a window of length 4*ms* centered around $t_{d}^{\mu }$, and no additional spike at any time. We define success loosely, because MPDP and FP-Learning do not converge onto generating the output spike exactly at $t_{d}^{\mu }$.

We train each network for a fixed number of learning blocks (10000 in the case of MPDP, 20000 for the others). Because we evaluate recall after each learning block, we can check whether the system has converged. We define capacity as the “critical load” *α*_90_, where on average 90% of the spikes are recalled after training. To approximate it, we plot the fraction of patterns correctly learned as a function of the load *α*. The critical load is defined as the point where a horizontal line at 90% correct recall meets the graph.

#### Testing noise tolerance

The threshold for LTD, *ϑ*_*D*_, is not only a way to impose homeostasis on the synaptic weights. It is also a safeguard against spurious spikes that could be caused by fluctuations in the input or membrane potential. The reason is that after convergence of weight changes for known input patterns the voltage mostly stays below *ϑ*_*D*_ for all non spike times due to the repulsion of the membrane potential away from threshold. This leaves room for the voltage to fluctuate without causing spurious spikes.

We apply noise in two conditions. First we want to know if a trained network is able to recall the learned input-output associations in the presence of noise, i.e. we train the network first and apply noise only during the recall trials. Second we test if a learning rule can be used to train the network in the presence of noise. In this condition, we test recall noise free.

We induce noise in two different ways. One way is to add a stochastic external current

$$\begin{array}{c} I_{ext}\left(t\right)=\frac{\sigma_{input}}{\sqrt{\tau_{m}}}\eta \left(t\right)\;. \end{array}$$ (12)

*η*(*t*) is a gaussian white noise process with zero mean and unit variance. The factor makes sure that the actual noise on the membrane potential has standard deviation *σ*_*input*_.

The other way is to jitter the input spike times. Instead of using presynaptic spike times $t_{i}^{\mu }$, we let the neurons spike at times

$$\begin{array}{c} {\hat{t}}_{i}^{\mu }=t_{i}^{\mu }+N\left(0,\sigma_{jitter}\right)\;, \end{array}$$ (13)

where N(0,σ) is a random number drawn from a gaussian distribution centered at zero with standard deviation *σ*.

If we apply noise only during recall, we use the weights after the final learning block and for each noise level *σ*_*input*, *jitter*_ we average the recall over 50 separate noise realizations and all training realizations.

Although both procedures lead to random fluctuations of the membrane potential in each pattern presentation, they lead to different results. The reason is that by using jitter on the input spike times, the statistics of the weights impact on the actual amount of fluctuations of the voltage. This has noticable consequences for the different learning rules.

### Learning algorithms used for quantitative comparison

Our goal is a quantitative analysis of the memory capacity of MPDP in the Chronotron task. We feel this necessitates a comparison to other learning rules. We chose ReSuMe [10], which is a prototypical learning rule for spiking output, E-Learning [6] as a powerful extension, and FP-Learning [11], which was shown to achieve optimal memory capacity in the task. Here, we provide a short description of all three rules.

#### The *δ*-rule, ReSuMe and the Pfister-rule

The *δ*-rule, also called the Widrow-Hoff-rule [19], lies at the core of a whole class of learning rules used to teach a neuronal network some target activity pattern. Synaptic changes are driven by the difference of desired and actual output, weighted by the presynaptic activity:

$$\Delta w\left(t\right)\propto f_{pre}\left(t\right)\left(f_{post}^{target}\left(t\right)-f_{post}^{actual}\left(t\right)\right)\;.$$ (14)

We denote pre- and postsynaptic firing rate with *f*_*pre*, *post*_. The target activity $f_{post}^{target}\left(t\right)$ is some arbitrary time dependent firing rate. The actual self-generated activity $f_{post}^{actual}\left(t\right)$ is given by the current input or voltage of the postsynaptic neuron (depending on the formulation), transformed by the input-output function *g*(*h*) of the neuron.

ReSuMe (short for Remote Supervised Method) is a supervised spike-based learning rule first proposed in 2005 [10]. It is derived from the Widrow-Hoff rule for rate-based neurons, applied to deterministic spiking neurons. Therefore, continuous time dependent firing rates get replaced with discrete spiking events in time, expressed as sums of delta-functions. Because these functions have zero width in time, it is necessary to temporally spread out presynaptic spikes by convolving the presynaptic spike train with a temporal kernel. Although the choice of the kernel is free, usually a causal exponential kernel works best. We also used ReSuMe with a PSP kernel to train Chronotron, but the results were worse than with the exponential kernel (data not shown). The weight change is given by

$$\begin{array}{c} \dot{w}\left(t\right)\propto \left[S_{d}\left(t\right)-S_{o}\left(t\right)\right]\left[a_{d}+\int_{0}^{\infty }\exp\left(-s/\tau_{plas}\right)S_{i}\left(t-s\right)ds\right]\;, \end{array}$$ (15)

where *S*_*d*_(*t*) is the desired, *S*_*o*_(*t*) is the self-generated and *S*_*i*_(*t*) the input spike train at synapse *i*. *τ*_*plas*_ is the decay time constant of the exponential kernel. *a*_*d*_ is a constant which makes sure that the actual and target firing rates match; learning also works without, therefore we choose *a*_*d*_ = 0 in our study. ReSuMe converges when both actual and desired spike lie at the same time, because in this case the weight changes cancel exactly.

In recent years, several rules for spiking neurons have been devised which can be viewed as an extension of the *δ*-rule to stochastic spiking neurons [12–15]. With the “PSP sum”

$$\begin{array}{c} \lambda_{i}=\sum_{k}\varepsilon \left(t-t_{i}^{k}\right)\;, \end{array}$$ (16)

the weight change takes the form

$$\begin{array}{c} {\dot{w}}_{i}\propto \left[S_{teacher}\left(t\right)-\rho \left(V\left(t\right)\right)\right]f\left(\rho \left(V\left(t\right)\right)\right)\lambda_{i}\left(t\right)\;. \end{array}$$ (17)

*S*_*teacher*_(*t*) is a stochastic realization of a given desired time dependent target firing rate, *ρ*(*V*(*t*)) is the instantaneous firing rate, which depends on the current membrane potential, and *f*(*ρ*) is a function which additionally scales the weight changes depending on the current firing rate. This rule was first derived by Pfister and colleagues in 2006. They started from the probability of a target spike train given the time course of the voltage and then computed the derivative of this probability with respect to the weights, which results in the weight change rule Eq 17.

Xie and Seung [13] derived a similar rule in a reward based framework, which exploits the correlation between fluctuations in the neuronal activity and a global reward signal. In particular, their rule uses the self-generated activity *S*_*out*_(*t*) instead of *S*_*teacher*_(*t*) in Eq 17. Clamping the output neuron to spike only at the desired spike times and keeping the reward positive and constant transforms their learning rule into the Pfister-rule.

#### E-Learning

E-Learning was conceived as an improved learning algorithm for spike time learning [6]. It is derived from the Victor-Pupura distance (VP distance) between spike trains [20]. The VP-distance is used to compare the similarity between two different spike trains. Basically, spikes can be shifted, deleted or inserted in order to transform one spike train into the other. Each action is assigned a cost, and the VP distance is the minimum transformation cost. The cost of shifting a spike is proportional to the distance it is shifted and weighted with a parameter *τ*_*q*_. If the shift is too far, it gets cheaper to delete and re-insert that spike.

E-Learning is a gradient descent on the VP distance and has smoother convergence than ReSuMe. In this rule, first the actual output spike train is compared to the desired spike train. With the VP algorithm it is determined if output spikes must be shifted or erased or if some desired output spike has no close actual spike so a new spike has to be inserted. Based on this evaluation, actual and desired spikes are put in three categories:

- Actual output spikes are “paired” if they have a pendant, i.e. a desired spike close in time and no other actual output spike closer (and vice versa). These spikes are put into a set *S*.
- Unpaired actual output spikes that need to be deleted are put into the set *D*.
- Unpaired desired output spike times are put into the set *J*, i.e. the set of spikes that have to be inserted.

To clarify, *S* contains pairs of “paired” actual and desired spike times, *D* contains the times of all unpaired actual spikes, and *J* the times of unpaired desired spike times. With the PSP sum as above, the E-Learning rule is then

$$\begin{array}{c} \Delta w_{i}=\gamma \left[\sum_{t_{ins}\in J}\lambda_{i}\left(t^{ins}\right)-\sum_{t^{del}\in D}\lambda_{i}\left(t^{del}\right)+\frac{\gamma_{r}}{\tau_{q}^{2}}\sum_{(t^{act},t^{des})\in S}\left(t^{act}-t^{des}\right)\lambda_{i}\left(t^{act}\right)\right]\;. \end{array}$$ (18)

*γ* is the learning rate, and *γ*_*r*_ is a factor to scale spike shifting relative to deletion and insertion.

The former two terms of the rule correspond to ReSuMe, except the kernel is not a simple exponential decay. The advantage of E-Learning is that the weight changes for spikes close to their desired location are scaled with the distance, which improves convergence and consequentially memory capacity.

#### FP-Learning

FP-Learning [11] was devised to remedy a central problem in learning rules like ReSuMe and others. Any erroneous or missing spike “distorts” the time course of the membrane potential behind it compared to the desired final state. This creates a wrong environment for the learning rule, and weight changes can potentially be wrong. Therefore, the FP-Learning algorithm stops the learning trial as soon as it encounters any spike output error. Additionally, FP-Learning introduces a margin of tolerable error for the desired output spikes. An actual output spike should be generated in the window of tolerance [*t*_*d*_ − *ϵ*, *t*_*d*_ + *ϵ*] with the adjustable margin *ϵ*. Weights are changed on two occasions:

1. If a spike occurs outside the window of tolerance for any *t*_*d*_ at time *t*_*err*_, then weights are depressed by Δ*w*_*i*_ ∝ − *λ*_*i*_(*t*_*err*_). This also applies if the spike in question is the second one within a given tolerance window.
2. If *t* = *t*_*d*_ + *ε* and no spike has occured in the window of tolerance, then *t*_*err*_ = *t*_*d*_ + *ε* and Δ*w*_*i*_ ∝ *λ*_*i*_(*t*_*err*_).

In both cases, the learning trial immediately ends, to prevent that the “distorted” membrane potential leads to spurious weight changes. Because of this property, this rule is also referred to as “First Error Learning”.

### Parameters of the simulations

#### Conductance based neuron

The parameters used are as follows: *C*_*m*_ = 0.25*nF*, *g*_*L*_ = 20*nS*, *V*_*L*_ = − 70*mV*, *V*_*thr*_ = − 50 *V*_*ex*_ = − 40*mV*, *V*_*h*_ = *V*_*reset*_ = *V*_*in*_ = − 75*mV*, Δ*g*_*s*_ = 0.001, Δ*g*_*f*_ = 0.04, *τ*_*f*_ = 3*ms*, *τ*_*s*_ = 12.5*ms*, and *τ*_*syn*_ = 3*ms*. The size of the neuronal populations is *N*_*i*_ = 142 and *N*_*e*_ = 571. For the MPDP rule, the parameters are: $\vartheta_{D}^{I}=-70mV$, $\vartheta_{P}^{I}=-52mV$, *γ* = 100, *η* = 5⋅10^−8^ and *τ*_*m*_ = *C*_*m*_/*g*_*L*_ = 12.5*ms*.

#### Simple LIF neuron

The neurons parameters are *τ*_*s*_ = 3*ms*, *τ*_*m*_ = 10*ms* and *V*_*thr*_ = 20*mV*. The reset potential is *V*_*reset*_ = − 5*mV* with MPDP and *V*_*reset*_ = 0*mV* for the other learning rules. For MPDP we use *ϑ*_*D*_ = 18*mV*, *ϑ*_*P*_ = 0*mV*, *γ* = 14, and *η* = 5⋅10^−4^. With ReSuMe, we find *τ*_*plas*_ = 10*ms*, and *η* = {10, 4, 2, 1}⋅10^−10^ for 200, 500, 1000 and 2000 neurons as good parameters. FP-Learning has only a single free parameter, the learning rate *η* = 10^−9^.

#### Numerical procedures

All networks with MPDP were numerically integrated using a simple Euler integration scheme. The simulations for the conductance based LIF neuron were written in Python and used a step size of 0.025 ms. The neurons parameters are set to values that are both biologically realistic and similar to those of the quantitative analysis. For reference, we put them into the S1 Complete Code.

The simulations of the simple neuron and scripts for analysis were written in Matlab (Mathworks, Natick, MA). Here, we used a step size of 0.1 ms.

The networks that were trained with ReSuMe, E-Learning and FP-Learning were simulated using an event-based scheme [21], since in these rules the subthreshold voltage is not important.

The parameters like learning rates and thresholds we use are set by hand for all plasticity rules. Before doing the final simulations, we did a search in parameter space by hand to find combinations which yield high performance in the Chronotron task.

The error we report in the figures throughout this article is the standard error of the mean (SEM) over all realizations.

## Results

In the following, we start with presenting our Membrane Potential Dependent Plasticity rule (MPDP). We constructed a simple yet biologically plausible feed-forward network and show that MPDP, when tested with spike pairs, is equivalent to inhibitory Hebbian STDP as reported by Haas and colleagues [16]. We then show that with MPDP the output neuron of this example can be trained to generate spikes at specific times. Lastly, we turn to a simplified model to evaluate and compare with other rules the attainable memory capacity with MPDP, as well as its noise tolerance.

### Membrane Potential Dependent Plasticity

We formulated a basic homeostatic requirement on the membrane potential of a neuron. The neuron should stay in a sensible working regime; in other words, its voltage should be confined to moderate values. We formalized this by introducing two thresholds on the voltage. The upper threshold *ϑ*_*D*_ lies between the firing threshold and the resting potential, and the lower threshold *ϑ*_*P*_ is equal to the resting potential. They define the bounds on the postsynaptic membrane potential *V*(*t*). Using these thresholds, we introduce an error function to measure the deviation of the membrane potential, see Eq 5 in Methods. Using a simple LIF neuron model with linear dynamics below the firing threshold, we computed an update rule for the weights:

$$\begin{array}{c} {\dot{w}}_{i}=\eta \left(-\gamma {\left[V\left(t\right)-\vartheta_{D}\right]}_{+}+{\left[\vartheta_{P}-V\left(t\right)\right]}_{+}\right)\sum_{k}\varepsilon \left(t-t_{i}^{k}\right)\;, \end{array}$$ (19)

where *η* is the learning rate, *γ* is a factor that scales depression relative to potentation, *ε*(*s*) is the kernel of the postsynaptic potentials, and $t_{i}^{k}$ is the time of the *k*th spike in input neuron *i*. Weight changes with this rule “bend” the voltage at the times of non-zero error towards the region between the two thresholds. Fig 1B shows how MPDP affects the voltage for recurring input activity.

### Homeostatic MPDP on inhibitory synapses is compatible with STDP

We first investigated the biological plausibility of a network with MPDP. Experimental studies on plasticity of cortical excitatory neurons often find Hebbian plasticity rules like Hebbian Spike Timing Dependent Plasticity (STDP; see [22–26] for examples). Reports on Anti-Hebbian plasticity or sensitivity to subthreshold voltage in excitatory cortical neurons are scarce [27–30]. However, it has been reported that plasticity in (certain) inhibitory synapses onto excitatory cells has a Hebbian characteristic [16], i.e. synapses active before a postsynaptic spike become stronger, those active after the spike become weaker. The net effect of this rule on the postsynaptic neuron is *Anti-Hebbian*, because weight increases tend to suppress output spikes. There are few other reports on inhibitory STDP [31], especially with a symmetric Hebbian window [32], which is implied to play a role in balancing excitation and inhibition in cortex [33].

In experimental investigations of STDP, neurons are tested with pairs of pre- and postsynaptic spikes. We mimicked this procedure in a simple network consisting of one pre- and one postsynaptic neuron, and one “experimentator neuron”. The postsynaptic neuron was modeled as a conductance based LIF neuron. The experimentator neuron has a fixed strong excitatory synaptic weight onto the postsynaptic neuron, so that a spike of the experimentator neuron causes a postsynaptic spike. We used it to control the postsynaptic spike times. The presynaptic neuron is inhibitory and its weight is small compared to the experimentator, so that it has negligible influence on the postsynaptic spike time. We probed synaptic plasticity by inducing a pair of a pre- and a postsynaptic spike at times *t*_*pre*_ and *t*_*post*_, and vary *t*_*pre*_ while keeping *t*_*post*_ fixed. The resulting weight change of the inhibitory synapse as a function of timing difference is shown in Fig 1C. The shape of the function is in qualitative agreement with experimental results [16].

It is necessary to assume the presence of an “experimentator neuron”. The reason is that the shape of the STDP curve explicitely depends on the specifics of spike induction since the MPDP rule is sensitive only to subthreshold voltage. For example, using a delta-shaped input current would lead to a LTD-only STDP curve, since the time the voltage needs to cross the firing threshold starting from equilibrium is infinitely short.

### Homeostatic MPDP allows associative learning

At first glance, it might seem unlikely that a homeostatic plasticity mechanism can implement associative learning. If the membrane potential is close to firing threshold, implicating a high probability for spiking, inhibitory synapses get potentiated, suppressing the membrane potential if the input occurs again. If the membrane potential is below the resting state, which means that the neuron is quiescent, inhibitory synapses get depressed. Because of the effect on the membrane potential, we refer to this type of plasticity as “Anti-Hebbian” [28], since it seems to be opposed to Hebbian learning which states “those who fire together, wire together”. However, the neuronal dynamics shows somewhat stereotypic behavior before, during and after each spike. To induce a spike, the neuron needs to be depolarized up to *V*_*thr*_, where active feed-back processes kick in. These processes cause a very short and strong depolarization and a subsequent undershoot of the membrane potential (hyperpolarization), from where it relaxes back to equilibrium [34].

**Fig 2:**
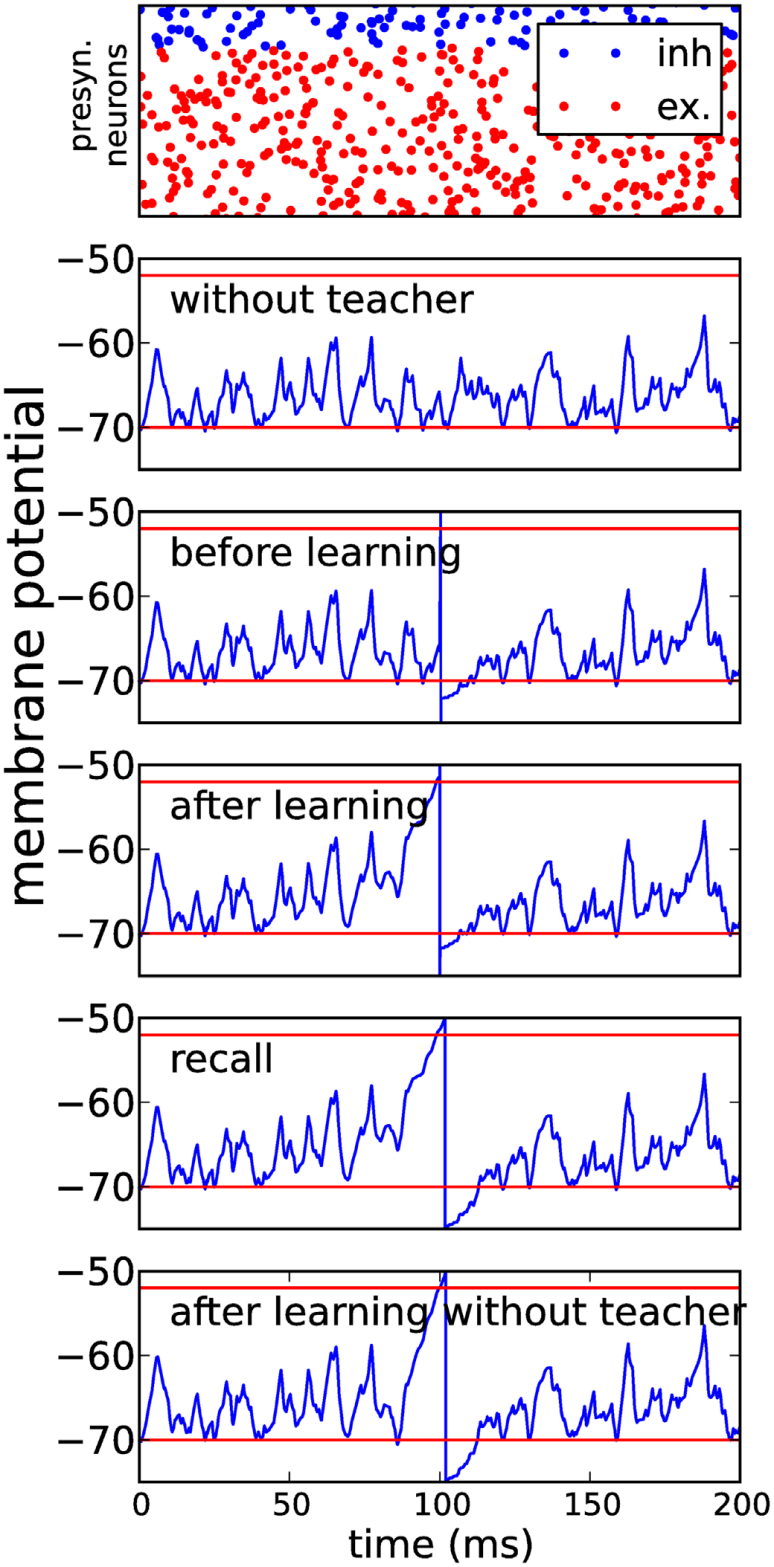
Hebbian learning with homeostatic MPDP on inhibitory synapses. A conductance based integrate-and-fire neuron is repeatedly presented with a fixed input pattern of activity in presynaptic inhibitory or excitatory neuron populations (top row—blue dots are excitatory, red dots are inhibitory spike times). The number of input neurons is *N*_*i*_ = 142 for the inhibitory population and *N*_*e*_ = 571 for the excitatory population. Second row shows the membrane potential before learning. The upper red line is the threshold for potentation of inhibitory synapses $\vartheta_{P}^{I}$, lower red line is resting potential and threshold for depression $\vartheta_{D}^{I}$. The third row shows the voltage as before with added teacher input by an additional population of excitatory neurons; this input induces a spike at *t* = 100*ms*. The fourth row shows the voltage after 100 learning steps with MPDP on inhibitory synapses only, with teacher input, the next row shows the recall without teacher. The spike is almost at the same position in the recall case. The last row shows the voltage after 1000 recall trials during which the inhibitory synapses were allowed to change under the MPDP rule. Despite this, the output spike is still close to the desired time, which shows that the output is approximately stable.

To demonstrate the capability of MPDP for learning of exact spike times, we constructed a simple yet plausible feed-forward network of *N*_*i*_ inhibitory and *N*_*e*_ excitatory neurons. Synaptic weights were initialized randomly. Both populations projected onto one conductance based LIF neuron. We presented this network frozen poissonian noise as the sole presynaptic firing pattern (Fig 2, top). Excitatory synapses were kept fixed and inhibitory synapses changed according to MPDP. First we let the network learn to balance all inputs from the excitatory population such that the membrane potential mostly stays between the upper threshold $\vartheta_{P}^{I}$ and the lower threshold $\vartheta_{D}^{I}$. We then introduced the teacher input as a strong synaptic input from a different source (e.g. a different neuron population, Fig 2, second to top). Balancing the voltages before actual training improves the reliability of spike generation by the teacher and therefore facilitates learning. After repeated presentations of the input pattern with the teacher input, inhibition around the teacher spike is released such that after learning the output neuron will spike close to the desired spike time even without the teacher input (Fig 2, third and fourth to top). Due to the sterotypical shape of the membrane potential around the teacher spike, a homeostatic learning rule is able to perform associative learning by release of inhibition.

**Fig 3:**
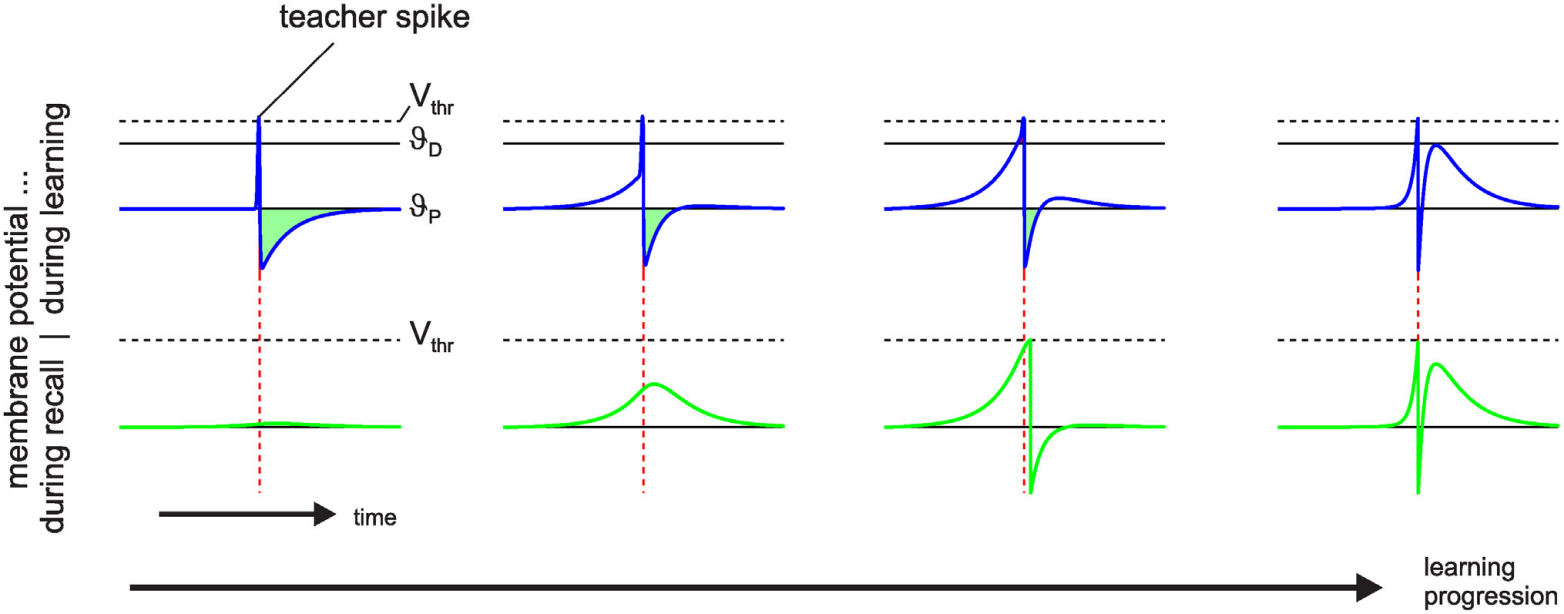
Hebbian learning with homeostatic MPDP. A postsynaptic neuron is presented the same input pattern multiple times, alternating between teaching trials with teacher spike (blue trace) and recall trials (green trace) to test the output. Initially, all weights are zero (left). The green area between the voltage and threshold for potentiation *ϑ*_*P*_ signifies the total amount of potentiation, similarly the red area between voltage and *ϑ*_*D*_ for depression; the latter is only visible in the second to right panel. Learning is Hebbian initially until strong depolarization occurs (second to left). When the spike first appears during recall, it is still not at the exact location of the teacher spike (second to right). Continued learning moves it closer to the desired location. Also, the time windows of the voltage being above *ϑ*_*D*_ and below *ϑ*_*P*_ shrink and move closer in time (right). Synaptic plasticity almost stops. The number of learning trials before each state is 1, 16, 53, and 1600 from left to right.

To further investigate the learning process, we simplified the setup. Instead of only inhibitory synapses being subject to plasticity, from now on all synapses were subject to MPDP. Therefore, crossing of the upper threshold *ϑ*_*D*_ induces a decrease of synaptic weights, while hyperpolarization below the threshold *ϑ*_*P*_ induces potentation of synaptic weights. All weights are allowed to change their sign. A population of *N* presynaptic neurons fires one spike in each neuron at equidistant times. They project onto a single postsynaptic LIF neuron and all weights are zero initially. In each training trial an external delta-shaped suprathreshold current is induced at the postsynaptic neuron at a fixed time relative to the onset of the input pattern (teacher spike). The postsynaptic neuron reaches its firing threshold instantaneously, spikes and undergoes reset into a hyperpolarized state (blue trace on the left in Fig 3). This is mathematically equivalent to adding a reset kernel at the time of the external current [11]. Because we set *ϑ*_*P*_ = *V*_*eq*_ = 0, potentiation is induced in all synapses which have temporal overlap of their PSP-kernel with the hyperpolarization. If the input pattern is presented a second time without the external spike the membrane potential shows a small bump around the time of the teacher spike. We continued to present the same input pattern, alternating between teaching trials (with teacher spike) and recall trials without teacher and with synaptic plasticity switched off. Plasticity is Hebbian until the weights are strong enough such that there is considerable depolarization before the teacher spike, inducing synaptic depression. Also, spike after-hyperpolarization is partially compensated by excitation, which reduces the window for potentiation. Continuation of learning after the spike association has been achieved (second to right plot) shrinks the windows for depression and potentiation, until they are very narrow and very close to each other in time. Because synaptic plasticity is determined by the integral over the normalized PSP during periods of depolarization and hyperpolarization, depression and potentiation become very similar in magnitude for each synapse and synaptic plasticity slows down nearly to a stop. Furthermore, the output spike has become stable. The time course of the membrane potential during teaching and recall trials is almost the same (Fig 3 right).

### Quantitative evaluation of MPDP

In the following, we perform a quantitative assessment of the properties of MPDP, especially memory capacity and recall behavior under input noise. For comparison, we perform the same assessment using a broad range of contemporary supervised learning rules (ReSuMe, E-Learning and FP-Learning, see Methods). We find moderately decreased memory capacity but enhanced noise robustness with MPDP compared to the other learning rules.

#### Memory capacity

We numerically evaluated the capacity of MPDP to train a network to produce precise spike times using the simplified feed-forward network described above. To simplify comparing results, we constructed input patterns and desired output spike times using what we call the the Chronotron framework [6]. Input patterns have length *T* = 200*ms*, with one spike in each of the *N* input neurons. Spike times are uniformly distributed over the interval and drawn independently for each presynaptic neuron. The time of the one desired output spike is also drawn from a uniform distribution. To test the capacity, we generate networks of different sizes *N*, and train the networks with different loads *α* = *P*/*N*, where *P* is the number of input-output associations to be learned. We introduce the load parameter *α*, since the memory capacity usually scales with *N* [6, 11]. Networks are trained in semi-batch mode. In a learning trial, one of the *P* patterns is presented concurrently with the teacher signal and the weights are updated immediately afterwards. A learning block consists of the presentation of all *P* patterns in randomized order. After convergence of training, we define the capacity as the load *α*_90_ where still 90% of patterns are recalled without error (i.e. failing to generate the desired spike or additional spurious spikes).

**Fig 4:**
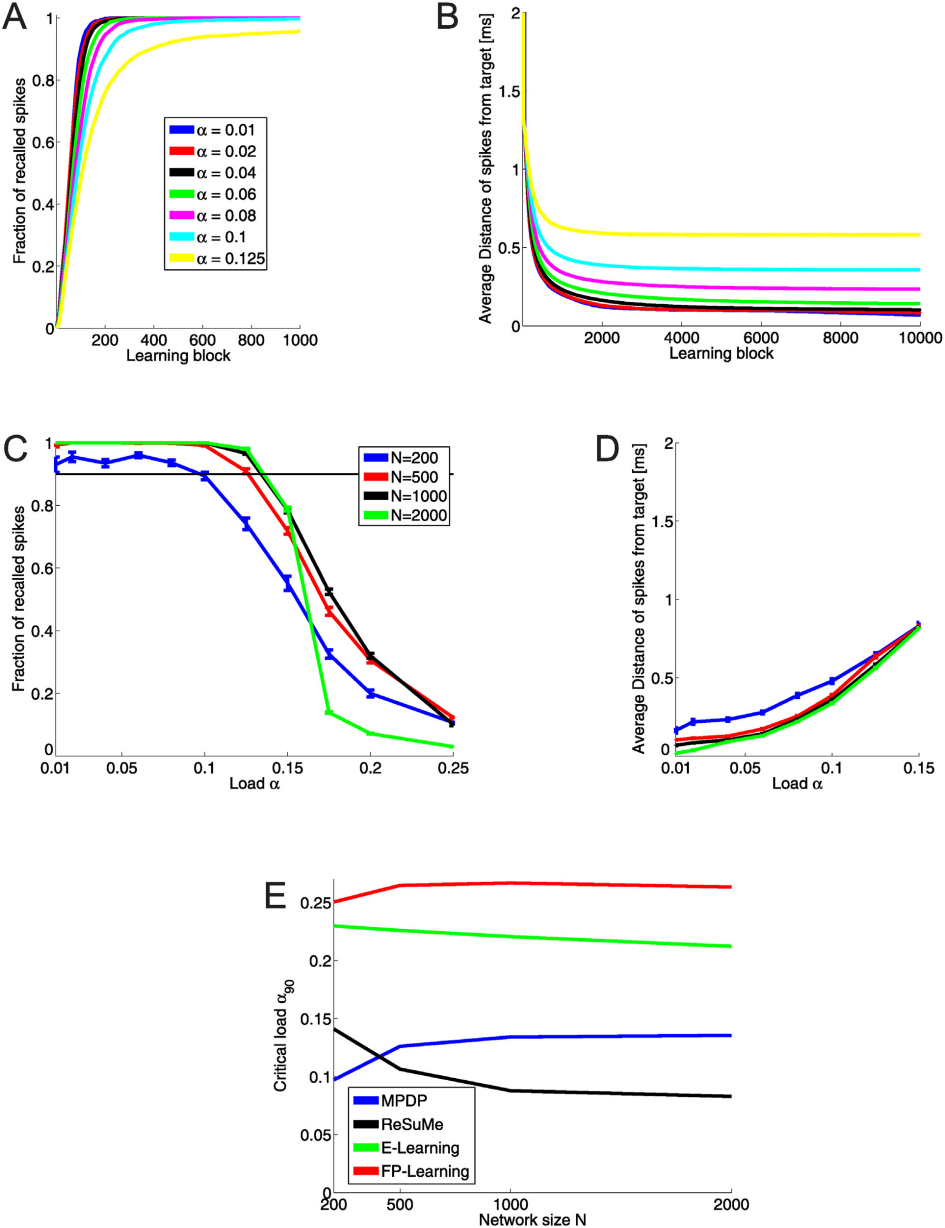
Capacity of networks with MPDP. **A:** Fraction of pattern where the network generates an output spike within 2 ms distance of target time $t_{d}^{\mu }$, and no spurious spikes. Network size is *N* = 1000. The desired spikes are learned within ≈ 600 steps. **B**: Average distance of output spikes to target for the same network size. Training continues even though the desired spikes are generated; however, they are pushed closer to the desired time. **C**: Average fraction of recalled spikes after 10000 learning blocks for all network sizes as a function of the load. Networks with *N* = 200 have a high probability to not be able to recall all spikes even for low loads. Otherwise, recall gets better with network size. The thin black line lies at fraction of recall equal to 90%. The critical load *α*_90_ is the point where the graph crosses this line. **D**: Average distance of recalled spikes as a function of the load. The lower the loads, the closer the output spike are to their desired location. **E**: Critical load as a function of network size for all four learning rules. MPDP reaches approximately half of the maximal capacity.

During training, we monitored the success of recall over time. The network of size *N* = 1000 generates the desired output spikes within the window of tolerance after 600 learning blocks (Fig 4A). However, weights are still changed by training, and continuation of it reduces the difference of actual and desired output spike time (see Fig 4B). After around 2000 learning blocks the average temporal error of all recalled spikes stays constant for the remainder of training. For *α* ≤ 0.1 the self-generated output spike is on average less than 0.5 ms away from the desired time. The final fraction of recalled spikes and average distance are shown in Fig 4C and 4D. The smallest network (*N* = 200) never reaches perfect recall, but has a capacity of *α*_90_ = 0.095. All other networks achieve perfect recall up to a load of *α* = 0.1 and a capacity of *α*_90_ ≈ 0.135. The average distance of spikes from teacher grows with the load, but stays below 0.5 ms.

We also checked if the memory capacity in terms of the length of learnable patterns with constant output spike rate is the same, compared to the simpler Chronotron setting. For the input neurons we generated poissonian spike trains with rate *r*_*in*_ of length *T* = (*αN*)/*r*_*out*_, where *αN* = *P* is the number of output spikes we want to imprint, and *r*_*out*_ = 5*Hz* = *r*_*in*_ is the average output firing rate. We then generated a target spike train with exactly *P* spike times by successively inserting spike times, while ensuring a minimal distance of 20*ms* between target spike times. We trained and checked success the same as above, and found no significant difference between the capacities in both conditions (data not shown).

To put these results into perspective, we trained Chronotrons again using three other learning rules, ReSume [10], E-Learning [6], and FP-Learning [11], and computed the respective memory capacity. Fig 4 shows the capacity of all plasticity rules. The upper bound established by FP-Learning is *α*_90_ ≈ 0.26. MPDP is capable of storing half of the maximal possible number of associations in the weights.

#### Training and recall with noise on the membrane potential

**Fig 5:**
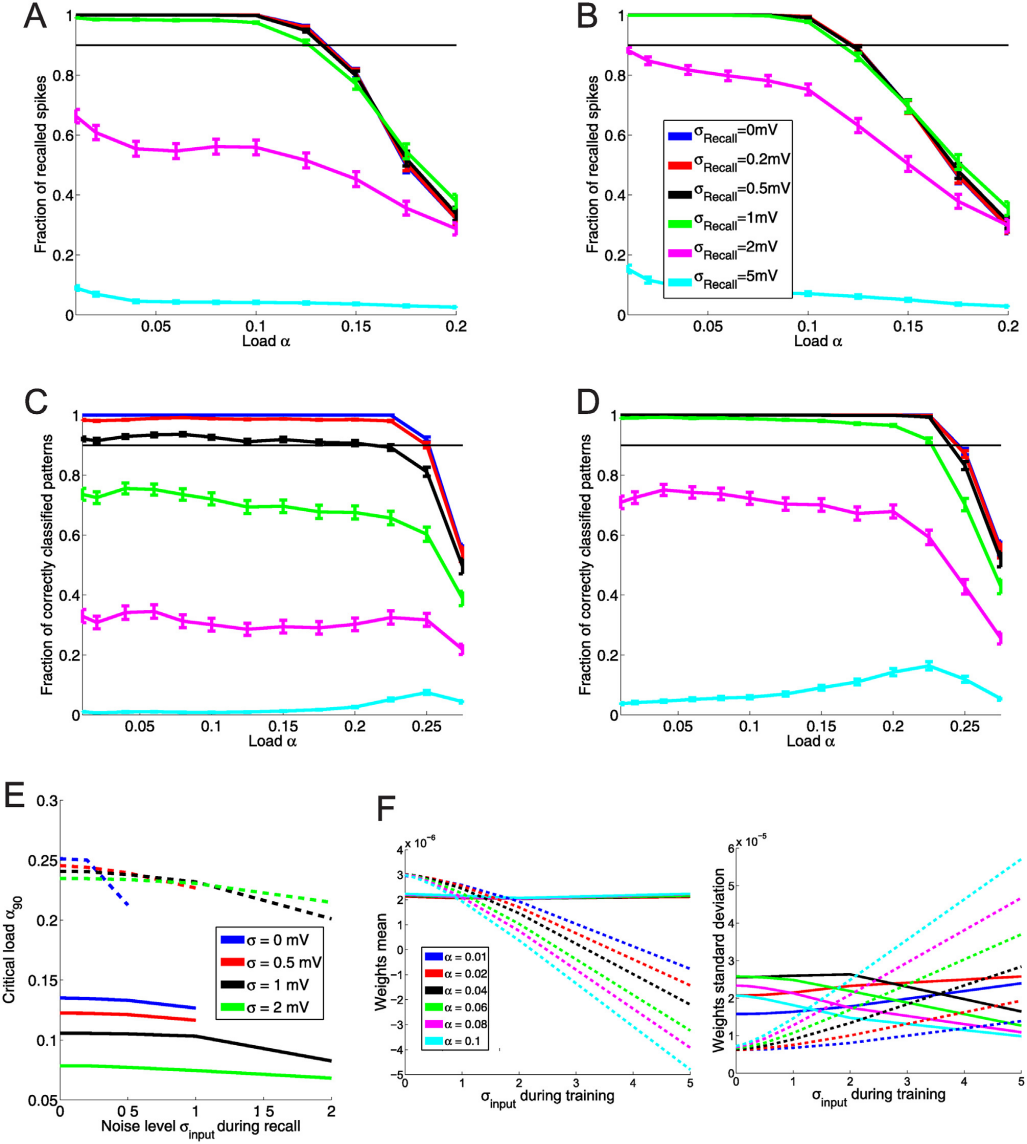
Capacity of networks under input noise. All network are of size *N* = 1000. **A**: Recall as a function of the load for different levels of noise during recall. Noise is imposed as an additional stochstastic external current. Networks were trained with MPDP. Up to a noise level *σ*_*input*_ = 1*mV* during recall, there is almost no degradation of capacity. **B**: Same as A, but with stochastic input noise of width 0.5*mV* during network training. The capacity is slightly reduced, but resistance against noise is slightly better. **C** and **D**: Same as A and B, but the network was trained with FP-Learning. The capacity is doubled. However, the network trained without noise shows an immediate degradation of recall with noise. If the network is trained with noisy examples (D, *σ*_*input*_ = 0.5*mV*), also recall with noise of the same magnitude is perfect. **E**: Comparison of capacity of networks trained with MPDP and FP-Learning depending on input noise during training and recall. Solid lines: MPDP, dashed lines: FP-Learning. Lines that are cut off indicate that the network failed to reach 90% recall for higher noise. x-axis is noise level during recall. Different colors indicate noise level during training. Curiously, although FP-Learning suffers more from higher noise during recall than during training, the capacity drops less than with MPDP. **F**: Comparison of weight statistics of MPDP (solid lines) and FP-Learning (dashed lines) after learning. Left plot is the mean, right plot is the standard deviation. With MPDP, the weigths stay within a bounded regime, the mean is independent of noise or load during training; the cyan line for *α* = 0.1 occludes the others. FP-Learning rescales the weights during training with noise: The mean becomes negative, and the standard deviation grows approximately linearly with noise level. This effectively scales down the noise by stochastic input.

Next, we turned to an evaluation of memory under the influence of noise. Having a noise free network is a highly idealized situation and neurons in the brain are more likely to be subject to noise, be it because of inherent stochasticity of spike generation or the fact that sensory inputs are almost never “pure”, but likely to arrive with additional more or less random inputs. First, we tested training and recall of spike times using an additional random input current of a given variance *σ* on the postsynaptic neuron. The random input is a gaussian white noise process with zero mean, and because inputs decay with the membrane time constant, this results in a additional random walk with a restoring force. We chose the variance of the input current to result in a random walk on the membrane potential with width *σ*_*input*_ ∈ {0, 0.2, 0.5, 1, 2, 5}*mV*. The width is the standard deviation of the random walk after time *τ*_*m*_. Afterwards, we evaluated the critical load of networks of size *N* = 200, 500, 1000 depending on the noise level during training and during recall. The results are shown in Fig 5.

**Fig 6:**
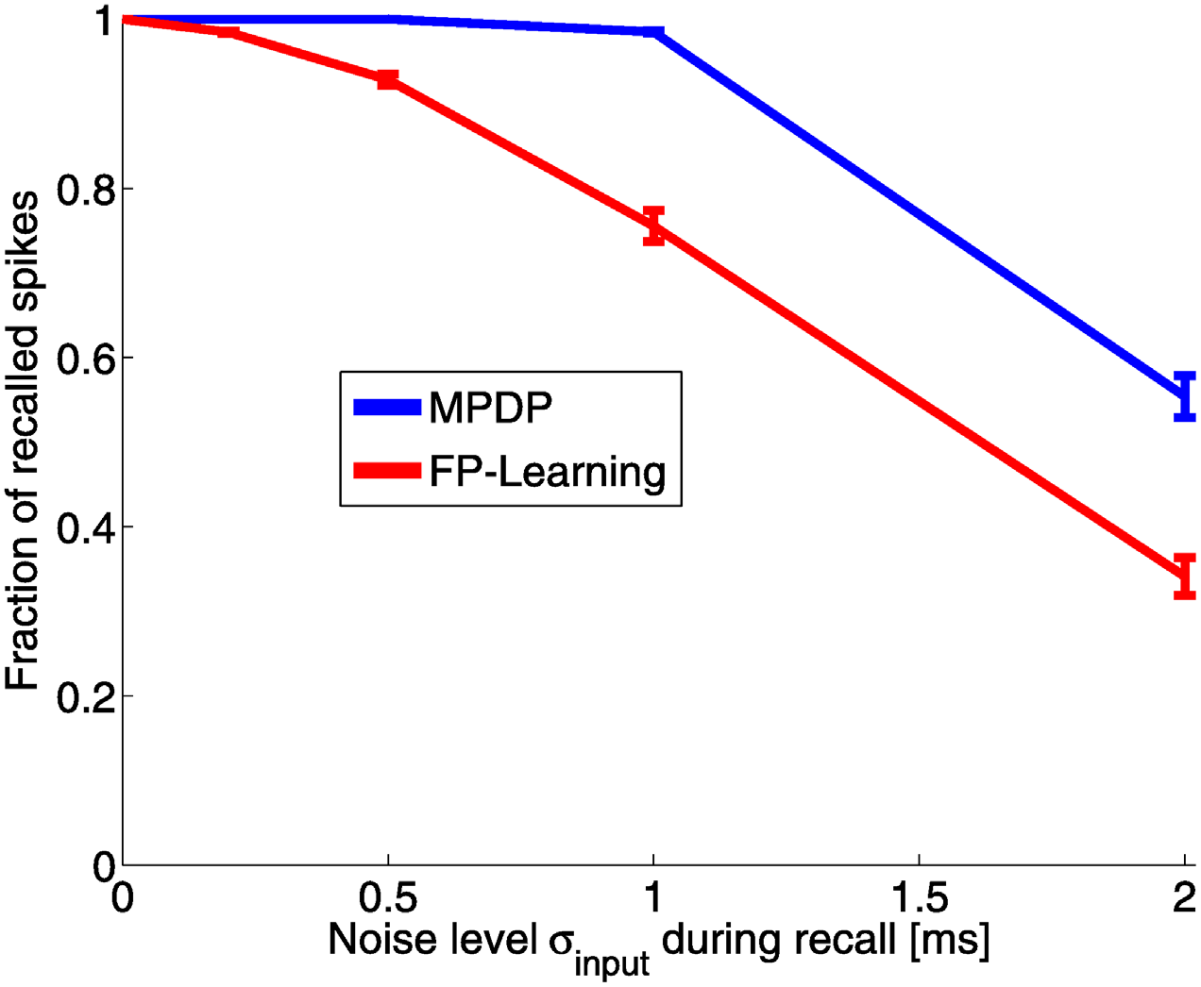
Fraction of recalled spikes under input noise. This plot shows the fraction of recalled spikes after learning as a function of the input noise during recall for load *α* = 0.04 and network size *N* = 1000. The network trained with MPDP perfectly recalls up to *σ*_*input*_ = 0.5*mV*, and with a slight drop for *σ*_*input*_ = 1*mV*. With the network trained with FP-Learning, there is a drop of the fraction of recalled spikes even with slight noise.

With MPDP, the network trained without noise can perfectly recall patterns up to a load of *α* = 0.1 even with additional noise input of *σ*_*input*_ = 0.5*mV* (Fig 5A). Adding noise during training decreases the capacity, but at the same time recall robustness against noise is improved (Fig 5B). This is contrasted by the network trained with FP-Learning. Here as well with ReSuMe (not shown), noise-free training results in a network with imperfect recall under noise (Fig 6). However, noise during training alleviates this problem. Training with a given noise width *σ*_*input*_ makes recall with the same and less noise width perfect. One interesting observation is that unlike with MPDP, with FP-Learning the memory capacity for noise-free recall stays constant regardless of noise during training (Fig 5C and 5D). This is explained by the variance of the weights after training. With FP-Learning, the variance increases approximately linearly with noise width, while the mean of the weights decreases linearly into negative values (Fig 5F). The resulting membrane potential is strongly biased towards hyperpolarized states. What FP-Learning effectively does during training is to scale up the weights, which scales down the noise in comparison. This reduces the influence of noise, but also leads to a membrane potential that stays below resting potential most of the time during input activity. Because of the threshold for LTP, MPDP can not scale the weights freely, therefore it suffers from a declining memory capacity.

#### Training and recall with input spike time jitter

As a second noise condition we tested training and recall in the case that the input spike times are not fixed. In each pattern presentation, we added to each presynaptic spike time some random number drawn from a gaussian distribution with mean zero and some given variance. The input is not frozen noise anymore, but a jittered version of the underlying input pattern $\left\{t_{i}^{\mu }\right\}$. Similarly to the condition of a stochastic input current, we tested the capacity of the network if during recall the input pattern are jittered or if during training the input is jittered (but noise free during recall).

**Fig 7:**
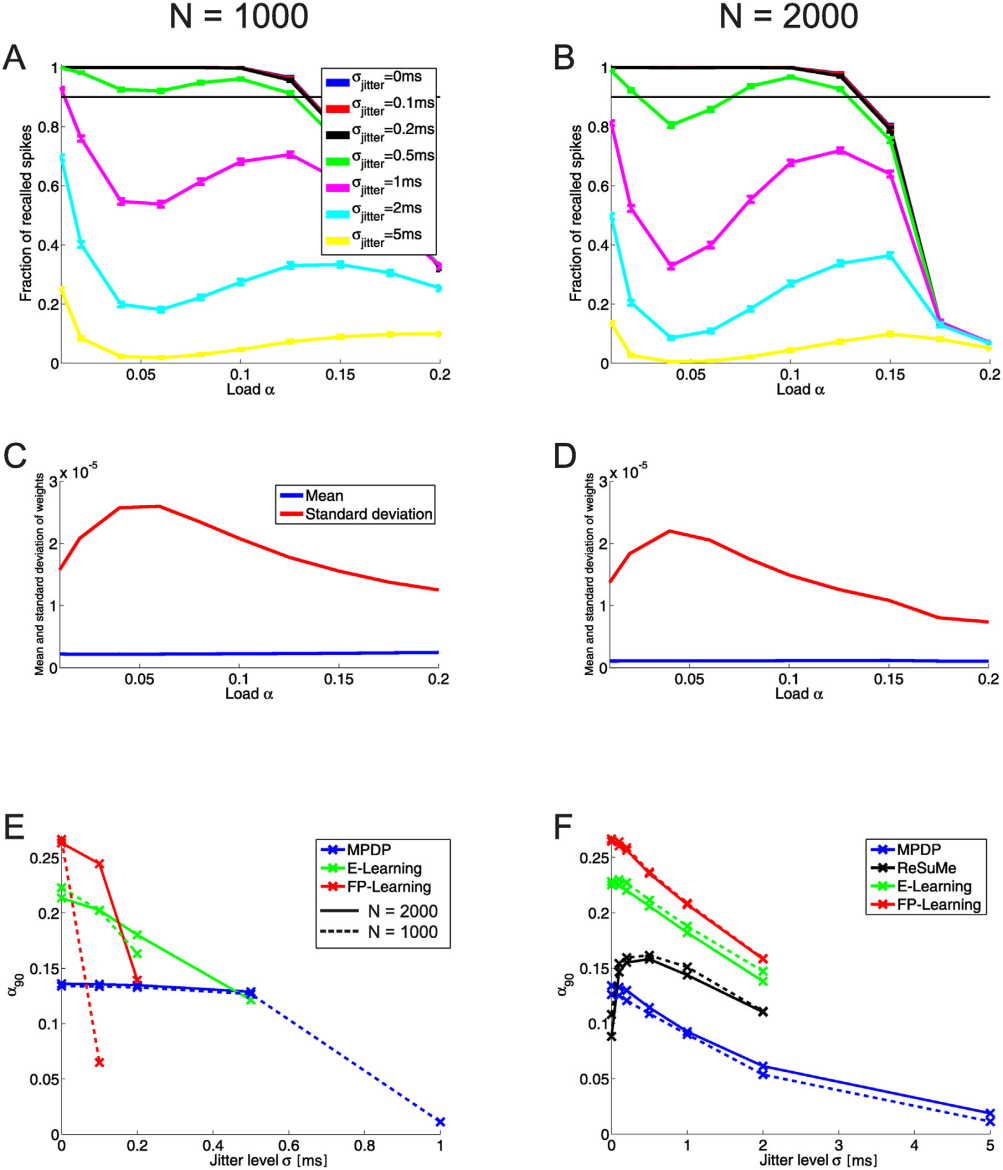
Recall and capacity with input jitter. **A:** Recall of networks trained noise-free with MPDP if during recall the input patterns are jittered (*N* = 1000). The black line lies on top of the blue and red ones (same in B). Up to *σ*_*jitter*_ = 0.5*ms*, the recall is unhindered. A curious feature is a “slump” in the recall for strong input jitter and intermediate loads. This slump is even more visible for the larger network with *N* = 2000 (**B**). The slump strongly correlates with the variance of the weights as a function of network load (**C** for *N* = 1000, **D** for *N* = 2000). The mean of the weights stays almost constant. **E:** Critical load as a function of input jitter during recall. The networks are trained noise free with different learning rules. Solid lines show *N* = 2000, dashed lines *N* = 1000. Crosses show sampling points. If a line is discontinued, this means that for this input jitter the networks do not reach 90% recall anymore. Recall for MPDP stays almost constant until *σ*_*jitter*_ = 0.5, while for the other learning rules a considerable drop-off of recall is visible. **F:** Noise free recall of networks trained with noisy input. For MPFP, E-Learning and FP-Learning alike the capacity drops with increasing training noise. The exception is ReSuMe. Here, the capacity strongly *increases* if the noise is small.

Fig 7A (*N* = 1000) and B (*N* = 2000) show the recall of networks trained noise free with MPDP if during recall the spike times of the input patterns are jittered. For jitter with a small variance (*σ*_*jitter*_ < 0.5*ms*), the recall is almost unaffected. For stronger jitter, recall deteriorates. A rather strange feature of the recall is that for intermediate loads *α* ≈ 0.05 the recall is worse than for loads close to the maximal capacity (*α*_90_ ≈ 0.125). This observation is counter-intuitive and calls for explanation, because recall usually becomes worse for memory systems if their load is close to the capacity. In our particular case, fluctations of the membrane potential due to jitter in the input spike times are scaled by the weights. This separates this noise condition from the one with stochastic input current. A comparison of the weight statistics of networks trained with MPDP after training shows that the slump in the recall covaries with the weight variance (Fig 7C and 7D). For *N* = 1000 the minimum of the slump lies at *α* = 0.06, which coincides with the maximum of the weight variance. For *N* = 2000, both lie at *α* = 0.04 instead. The mean of the weights does have little to no influence on that; it stays almost constant as a function of load. E-Learning and FP-Learning do not have the same characteristics (data not shown). For example, with FP-Learning weight average and variance stay basically constant until a load of *α* ≈ 0.2, rather close to the capacity. Only then the mean decreases and variance increases (see for example Fig 5F, right plot for *σ*_*input*_ = 0 during training).

Networks trained without noise and tested with jittered input show a similar behavior to noise induced by an external stochastic current (Fig 5E, blue lines, versus Fig 7E). Networks trained with MPDP tolerate noise up to a certain degree without showing a deterioration of recall. With the other learning rules, the recall gets worse with arbitrary small noise levels. On the other hand, training a network with FP-Learning while injecting stochastic currents (the previous noise condition) led to almost unharmed capacity. The reason is that FP-Learning “downscales” the noise by scaling up the weight variance. This is not a viable path for jitter of input spike times. Therefore, E-Learning and FP-Learning as well as MPDP show a decrease of capacity if during training the input spike times are jittered. An interesting outlier is ReSuMe. The networks trained noise free with ReSuMe have low capacity and unstable recall. Even with slight jitter the recall does not reach 90% anymore. Therefore, we do not include ReSuMe in Fig 7E. However, training the network with jitter leads to an increase of capacity (Fig 7F).

## Discussion

Our goal was to develop a synaptic plasticity rule that can be used to train a neuron to generate temporally structured output in a biophysically plausible way. Specifically, we wanted to find a plasticity mechanism that does not need any supervisory error signal providing specific instructions on the necessary weight changes. Instead, we aimed for a teacher that tells the neuron when to spike, using a manipulation of the neuron with a clear biophysical interpretation, namely inducing strong suprathreshold currents. Additionally, the plasticity rule should be rooted in experimental findings. To this end we introduced a synaptic plasticity mechanism that is based on the requirement to balance the membrane potential and therefore uses the postsynaptic membrane potential rather than postsynaptic spike times as the relevant signal for synaptic changes (Membrane Potential Dependent Plasticity, MPDP). We have shown that this simple rule allows the somewhat paradoxical temporal association of enforced output spikes with arbitrary frozen noise input spike patterns (Chronotron). Before, this task could only be achieved with supervised learning rules that provided knowledge not only about the desired spike times, but also about the type of each postsynaptic spike (desired or spurious). With MPDP, the supervisor only has to provide the desired spike, while the synapse endowed with MPDP distinguishes between desired and spurios spikes exploiting the time course of the voltage around the spike. Additionally, the sensitivity of MPDP to subthreshold membrane potential allows for robustness against noise.

### Biological plausibility of MPDP

Spike-Timing-Dependent Plasticity (STDP) is experimentally well established and simple to formalize, which made it a widely used plasticity mechanism in modelling. It is therefore important to note that MPDP is compatible with certain experimental results on STDP, in particular with those of causal Hebbian STDP on inhibitory synapses [16]. The reason is that spikes come with a stereotypic trace in the membrane potential. The voltage rises to the threshold, the spike itself is a short and strong depolarization, and afterwards the neuron undergoes reset, all of which are signals for MPDP. Pairing a postsynaptic spike with presynaptic spikes at different timings gives rise to a plasticity window which shares its main features with the STDP window: The magnitude of weight change drops with the temporal distance between both spikes and the sign switches close to concurrent spiking.

It is known that the somatic membrane potential plays a role in synaptic plasticity. A few studies investigated the effect of prolonged voltage deflections by clamping the voltage for an extended time while repeatedly exciting presynaptic neurons (e.g. see [35]). However, MPDP predicts that synaptic plasticity is sensitive to the exact time course of the membrane potential, as well as the timing of presynaptic spikes. This necessitates that dendrites and spines reproduce the time course of somatic voltage without substantial attenuation. Morphologically the dendritic spines form a compartement separated from the dendrite, which, for example, keeps calcium localized in the spine. It has been a topic under investigation whether the spine neck dampens invading currents. Despite experimental difficulties in measuring spine voltage, recent studies found that backpropagating action potentials indeed invade spines almost unhindered [36]. Furthermore, independently of spine morphology and proximity to soma, the time course of a somatic hyperpolarizing current step is well reproduced in dendrites [37] and spines [38]. This shows that at least in principle the somatic voltage trace can be available at the synapse. In turn, voltage-dependent calcium channels can transform subthreshold voltage deflections into an influx of calcium, the major messenger for synaptic plasticity. A few studies found that short depolarization events act as signals for synaptic plasticity [27, 29], with a dependence of sign and magnitude of weight change on the timing of presynaptic spikes.

Another important point is the sign of synaptic change. “Membrane Potential Dependent Plasticity” per se is a very general term which potentially could include many different rules [39, 40]. In this study, MPDP serves as a mechanism that keeps the membrane potential bounded. For inhibitory synapses this requirement results in a Hebbian plasticity rule, which has been reported previously [16]. Inhibitory neurons in cortex have been implied to precisely balance excitatory inputs [41]. MPDP on excitatory synapses is necessarily “Anti-Hebbian”. Lamsa et al. [28] found that pairing presynaptic spikes with postsynaptic hyperpolarization can lead to synaptic potentiation, albeit on excitatory synapses onto inhibitory interneurons. This was caused by calcium permeable AMPA receptors (CP-AMPARs) present in these synapses. However, Anti-Hebbian plasticity does not rely on CP-AMPARs alone. Verhoog et al. [30] found Anti-Hebbian STDP in human cortex in excitatory synapses, which depends on dendritic voltage-dependent calcium channels. Taken together, these findings demonstrate the existence of cellular machinery which could implement homeostatic MPDP, either on excitatory or inhibitory synapses.

### Properties and capabilities of Homeostatic MPDP

We derived homeostatic MPDP from a balance requirement: Synapses change in order to prevent hyperpolarization and strong depolarization for recurring input activity. This kind of balance reduces metabolic costs of a neuron and keeps it at a sensible and sensitive point of operation [42]. The resulting plasticity rule is Anti-Hebbian in nature because synapses change to decrease net input when the postsynaptic neuron is excited and to increase net input when it is inhibited. However, spike after-hyperpolarization turns homeostatic MPDP effectively into Hebbian plasticity. Every postsynaptic spike causes a voltage reset into a hyperpolarized state. Therefore synapses of presynaptic neurons which fired close in time to the postsynaptic spike will change to increase net input if the same spatio-temporal input pattern re-occurs. The total change summed over all synapses depends on the duration and magnitude of hyperpolarization. Because the induced synaptic change reduces this duration, total synaptic change is also reduced. The same is true for total synaptic change to decrease net input, which depends on the duration where the membrane potential stays above *ϑ*_*D*_ (resp. $\vartheta_{P}^{I}$ for inhibitory synapses) and which reduces this duration in future occurances. If the rise time of the voltage before the spike and residual spike after-hyperpolarization are both short and close in time, potentiation and depression will approximately cancel around a spike.

In this view, synaptic plasticity or “learning” is the consequence of imbalance. A spike is stable if the time course of the voltage in its proximity leads to balanced weight changes. This means, if input is just sufficient to cause a spike, the voltage slope just before the spike is shallow and synaptic depression outweighs potentiation. On the other end of the scale, the delta-pulse shaped currents used to excite the postsynaptic neuron during Chronotron training are very strong inputs. They are not unlearned. Instead, the weights potentiate until the membrane potential is in a balanced state, and the neuron fires the teacher spike on its own when left alone.

Yet a different view is that the output neuron with MPDP achieves associative learning. In this setting, the teacher is an input channel that elicits a certain response, namely an output spike at a specific time. This response is transferred to another input channel, in our case the population of presynaptic neurons. This is the basic principle of associative learning as captured in theoretical models (e.g. ISO learning [43]).

Lastly, an interesting aspect of MPDP is the emergence of robustness against noise. Most obviously, with the choice of the threshold for depression the neuron sets a minimal distance of the voltage to the firing threshold for known input patterns. This allows to have perfect recall in the case of noisy input in the Chronotron. The second effect of the depression threshold is more subtle. Not only does it prevent spurious spikes, but through learning the slope of the membrane potential just before the desired spike tends to become steep. This is necessary to prevent spike extinction by noise. To see how this influences noise robustness, consider an output spike with a flat slope of the voltage. Increasing the voltage slightly around the spike time moves the intersection of the voltage with the firing threshold forward in time by a proportionally large margin. Decreasing voltage moves it backwards in time or could even extinguish the spike; a flat slope implies a low peak of the “virtual” membrane potential. MPDP in contrast achieves a state which is robust against spike extinction as well as the generation of spurious spikes. On the downside, keeping the voltage away from the firing threshold as well as imposing steepness on the slope just before spikes puts additional constraints on the weights. Robustness comes at the cost of capacity.

### Relation of MPDP to other plasticity rules

#### Voltage dependent plasticity rules

The idea of making a plasticity rule at least partially dependent on the voltage is not new. The plasticity rule devised by Shouval and colleagues is inspired by the BCM rule and makes use of the depolarization dependent unblocking of NMDA receptors to mechanistically retrieve the well-known Hebbian STDP window [40]. This plasticity rule is very complex involving many interactions between different quantities at the synapse and has little similarity to MPDP. In another plasticity rule developed by Clopath et al. [39], synaptic depression is computed at the time of presynaptic spikes, and its magnitude is given by ${\left[\bar{V}\left(t\right)-\theta_{-}\right]}_{+}$, where *θ*_−_ is a threshold on the voltage and $\bar{V}$ is a low-pass filtered version of the membrane potential. This term is very similar to how depression is computed in our MPDP rule. Synaptic potentiation is basically Hebbian, i.e. it depends on the correlation of presynaptic spiking and postsynaptic depolarization. The interplay of both contributions lead to a stabilization of weight changes in the network given some input activity. An interesting rule is the Convallis rule by Yger and Harris [44]. The Convallis rule and MPDP are derived from a similar objective but with opposite sign. Yger and Harris postulate the objective that the distribution of voltages should have peaks at extreme values, that means the neuron is preferrably hyperpolarized or close to (and above) the firing threshold. The resulting plasticity rule is a Hebbian MPDP rule which reinforces synapses that are active during postsynaptic depolarization and weakens those active during hyperpolarization. To maintain network stability, they have to introduce weight scaling. A network endowed with the Convallis rule in an unsupervised manner learns firing rate representations of input stimuli which can be read out by a linear classifier.

#### Supervised learning rules

There are many supervised learning algorithms that are used to train neuronal networks to generate desired spatio-temporal activity patterns. They either involve an explicit comparison of the self-generated output to the desired target activity, or rely on spike-like signals that do not impact the neuronal dynamics. Based on different characteristics, we broadly put them into three different classes. E-Learning and FP-Learning [6, 11] are examples of algorithms of the first class which are used to train a neuron to generate spikes at exactly defined times. They first observe the complete output and then evaluate it against the target. E-Learning performs a gradient descent on the Victor-Purpura distance [20] between both spike trains. This means that the weight changes associated with one particular spike (actual or desired) can depend on distant output spikes. This non-locality in time is hard to implement in a biological neuron, since at some instance (supervisor or directly at the synapse) past spike times have to be temporarily stored for comparison with future spike times. In FP-Learning, the training trial is interrupted if the algorithm encounters an output error. Subsequent spikes are not evaluated anymore. This necessitates an external supervisor shutting down plasticity after an error. While more plausible than E-Learning, such a mechanism might be difficult to realize in a biological neuron.

Another class of learning algorithms emerged recently with the examples PBSNLR([45], but see also [46]) and HTP [11]. They take an entirely different route. The postsynaptic membrane potential is treated as a static sum of PSP kernels weighted by the respective synaptic weight, similar to the Spike Response Model of the LIF neuron (see Methods, [17]). In PBSNLR, the membrane potential is sampled on a discrete set of points in time, including the desired spike times. Then, the weights are learned using the perceptron learning rule to ensure that the membrane potential stays below the firing threshold for non-spike times and above for desired spike times. HTP is a more sophisticated algorithm also employing the perceptron learning rule. The weights are projected into a subspace of the weight space to ensure threshold crossing from below at the desired times. Then, the algorithm dynamically samples the membrane potential at several points in time to ensure that the voltage stays below the firing threshold for non-spike times (also those that are not part of the sampled set). HTP has guaranteed convergence in finite time if a solution exists; this property is the basis to evaluate the maximal capacity in the spike time learning task [11]. These algorithms were devised as technical solutions and are very artificial. However, MPDP bears some similarity to the described procedure: Except close to teacher inputs, at every point in time recently active synapses get depressed if the voltage is above the threshold for depression. This is comparable to a perceptron classification on a continuous set of points.

A third class of algorithms compares actual and target activity locally in time. In contrast to the algorithms mentioned above, they are usually not used to learn exact spike times, but rather continuous time dependent firing rates. The ur-example is the Widrow-Hoff rule [10, 19]. More recently, similar rules were developed by Pfister et al. [12], Brea et al. [14] and Urbanzcik and Senn [15]. In the following, we call them the “Pfister-rule” in short. In contrast to the Widrow-Hoff rule, the Pfister-rule is defined for spiking LIF neurons with a “soft” firing threshold, i.e. spike generation is stochastic and the probability of firing a spike is a monotonous function of the current voltage. In the Pfister-rule, at every point in time the synaptic change is proportional to the difference of the current firing rate and a target firing rate specified by an external supervisor. When it comes to biological implementation, the central problem of this rule is the comparison of self-generated and target activity. It is derived from the abstract goal to imprint the target activity into the network. This target needs to be communicated to the neuron and synaptic plasticity has to be sensitive to the difference of the neurons’ own current acticity state (implicitely represented by its membrane potential) and the desired target activity. Usually, no plausible biological implementation for this comparison is given. The combination of homeostatic MPDP, hyperpolarization and a teacher now offers a solution to both problems. The teacher provides information about the target activity through temporally confined, strong input currents which cause a spike. Spike after-hyperpolarization allows to compare the actual input to the target without inducing spurious spikes detrimental to learning. The more spike after-hyperpolarization is compensated by synaptic inputs, the closer the self-generated activity is to the target and the less synapses need to be potentiated. This is implemented naturally in MPDP, where potentiation is proportional to the magnitude and duration of hyperpolarization. On the other hand, strong subthreshold depolarization implies that self-generated spurious spikes are highly probable, and weights need to be depressed to prevent spurious spikes in future presentations of this input activity.

A further solution for the problem of how information about the target is provided was given by Urbanczik and Senn [15]. Here, the neuron is modeled with soma and dendrite as separate compartements instead of point neurons as used in this study. The teacher is emulated by synaptic input projecting directly onto the soma, which causes a specific time course of the somatic membrane potential. The voltage in the dendrite is determined by a different set of synaptic inputs, but not influenced by the somatic voltage; however, the soma gets input from the dendrites. The weight change rule then acts to minimize the difference of somatic (teacher) spiking and the activity as it would be caused by the current dendritic voltage. This model represents a natural way to introduce an otherwise abstract teacher into the neuron. Nonetheless, the neuron still has to estimate a firing rate from its current dendritic voltage, for which no explicit synaptic mechanism is provided. Also, it is worth noting that the model of Urbanczik and Senn requires a one-way barrier to prevent somatic voltage invading the dendrites; in contrast, MPDP requires a strong two-way coupling between somatic and dendritic/synaptic voltage.

Another putative mechanism for a biological implementation of the *δ*-rule was provided by D’Souza et al. [47]. In this model, a neuron receives early auditory and late visual input. By the combination of spike frequency adaptation (SFA) and STDP, the visual input acts as the teacher that imprints the desired response to a given auditory input in an associative manner. However, the model is quite specific to the barn owl setting; for example, parameters have to be tuned to the delay between auditory and visual input.

Applying the Pfister-rule type to fully deterministic neurons can lead to unsatisfactory results. ReSuMe is an example of such a rule [10]. Its memory capacity is low, but it increases sharply if the input is noisy during training (see Fig 6). The explanation is that in a fully deterministic setting, the actual spike times do not allow a good estimation of the expected activity. This sounds paradoxial. But if we consider a deterministic neuron with noise-free inputs the membrane potential can be arbitrarily close to the firing threshold without crossing it. But even the slightest perturbation can cause spurious spikes at those times. This leads to bad convergence in Chronotron training, since the perturbations caused by weight changes for one pattern can easily destroy previously learned correct output for another pattern [11]. The problem of these rules is the sensing of the activity via the instantaneous firing rate. Therefore, the explicit sensitivity to subthreshold voltages of MPDP is advantageous if training examples are noise free.

### Conclusion

We propose Membrane Potential Dependent Plasticity as a viable mechanism for spike time learning in biological spiking neuronal networks. As we have shown, there already are several learning rules which achieve this goal. However, the existing learning rules in one way or another lack a plausible biological implementation regarding the signalling of the desired activity to the synapse by the supervisor and/or the computation of the error between desired and actual output activity. The main advantage of MPDP over the previous rules is the clear biological interpretation of the learning mechanism. The synaptic plasticity rule is founded on physiological findings, and it is geared with the teacher that induces strong currents to signal desired spike times.

It is often the case that including biolgical considerations worsens quantitative behavior like memory capacities of modelled systems, as it softens the mathematical rigor of technical learning rules that have been designed with optimality in mind [6, 18]. To show that MPDP is useful as a learning rule, we compared it with ReSuMe, E-Learning and FP-Learning. This comparison revealed the drawbacks of MPDP: The memory capacity is roughly half of the maximum and output spikes lag by a short period of time to the desired times. Also, switching associations after training a network is difficult. With a rule like FP-Learning, after training the desired output spikes can freely be redefined and subsequently learned, while output spikes learned with MPDP tend to stay fixed. On the other hand, using MPDP leads to networks that a robust against input noise even if training was noise-free, a feature that is unique to it. Taken together, the membrane potential dependent plasticity proposed here is a neurobiologically plausible mechanism that might explain abilities of neuronal networks in the brain to process temporally precise neuronal codes.

## Supporting Information

S1 Complete Code Zip-file containing scripts generating data and figures. In the supporting material, embedded in the appropriate folder structure we include our scripts written in Python and Matlab that generate the data used in the figures in this article. Further instructions are included within Readme.txt inside the zip-file.
(ZIP)
